# For Gonorrhœa

**Published:** 1877-12

**Authors:** 


For Gonorrhœa.—Dr. Leonard says: Quinine 12 grains; acid sulph. aromar., 15 grains; aquæ rosæ, 4 ounces. Ft. injectio. Wash out the urethra and use half an ounce of the fluid at a time, two or three times a day, retaining it in contact a short time. This is the best local treatment I have found. It does not preclude the usual antiphlogistic treatment during the inflammatory stage, but it will take the place of any or all of the specifics in this disease.

